# Awareness, use and future interest in cytisine for smoking cessation in adults who smoke or have quit within the past year in the UK: A mixed-methods study

**DOI:** 10.1016/j.abrep.2026.100726

**Published:** 2026-06-30

**Authors:** Megan Devereux, Jie Lyu, Vera Helen Buss, Sharon Cox, Lion Shahab, Dimitra Kale

**Affiliations:** a Department of Behavioural Science and Health, University College London, UK; b Behavioural Research, UK

**Keywords:** Cytisine, Stop smoking medication, Cytisinicline, Mixed methods, Smoking cessation, COM-B

## Abstract

**Background and aims:**

Cytisine is an effective smoking cessation pharmacotherapy introduced in the UK in 2024. Assessing awareness, use, and interest is essential to assess its potential role in smoking cessation. We examined cytisine awareness and use among people who smoked in the past year, and interest in and factors influencing future use among people who currently smoke.

**Methods:**

An explanatory sequential mixed-method design study conducted between April–July 2025 in the UK; 193 people reporting past-year smoking (mean-age = 37.7,SD = 13.4) completed an online survey, and 16 participated in semi-structured interviews. Logistic regression examined correlates of cytisine awareness and interest in future use. Interviews were analysed thematically using the COM-B framework to explore perceived barriers and facilitators to cytisine use.

**Results:**

Cytisine awareness was low (*n* = 43,22.3%). Among those with a past-year quit attempt (*n* = 141), 4.3% reported cytisine use. Among those who currently smoke (*n* = 169), 71.6% reported interest in future use. The most endorsed factors encouraging future use were confidence in cytisine's helpfulness (52.7%) and safety (46.7%). Awareness was associated with past-year quit attempts (aOR = 3.21,95%CI 1.09–9.51), and fewer cigarettes/day (aOR = 0.89,95% CI 0.80–0.98). Interest in future use was associated with past-year quit attempts (aOR = 3.88,95%CI 1.70–8.88). Interviews highlighted a need for more information on cytisine, concerns about its effectiveness, side effects and dosing, and the role of healthcare guidance and social influences in shaping interest in future use.

**Conclusions:**

Despite low awareness and use of cytisine, interest in future use is high. Addressing informational and practical barriers could support wider cytisine uptake.

## Highlights

• Awareness of cytisine was low, reported by 22% of participants in a self-selected convenience adult sample in the UK.
• Cytisine use during the most recent quit attempt was rare, with only 4% of recent quitters reporting use.
• Interest in trying cytisine in a future quit attempt was high, expressed by 72% of participants currently smoking.
• Key barriers of cytisine use included limited knowledge, dosing complexity, and stigma around medication use.
• Facilitators of cytisine use included perceived effectiveness, safety, and positive experiences from peers.

## 1 Introduction

Smoking remains a leading cause of preventable illness and premature death in the United Kingdon (UK) (Brown et al., 2018). It is a difficult behaviour to change, with high rates of relapse even after six months of abstinence (Hughes et al., 2008). Although structured behavioural support and pharmacotherapy are proven to help people stop smoking (Cahill et al., 2013; Hartmann-Boyce et al., 2018; Office for Health Improvements & Disparities, 2023; Stead & Lancaster, 2012), many do not make use of these interventions (Jackson, Brown, et al., 2025), making long-term abstinence difficult to achieve (Hughes et al., 2008; Jackson, Brown, et al., 2025). Among the available pharmacotherapies, varenicline and cytisine are the most effective options currently available in the UK (Lindson et al., 2023). As cytisine has only recently become available in the UK (NHS, 2024), there is limited empirical evidence describing its real-world awareness and use following introduction. Understanding awareness, uptake, future intention to use and factors associated with its use is important for assessing its potential contribution to smoking cessation support in the UK.

Cytisine is a plant alkaloid (Tutka et al., 2019) that has been used as a smoking cessation aid for several decades in Central and Eastern Europe (Etter et al., 2008). It acts as a partial agonist at the α4β2 nicotinic acetylcholine receptor, similar to varenicline, meaning that it mimics nicotine and competes with it for receptor binding (Tutka et al., 2019). This reduces the rewarding effects of smoking and withdrawal symptoms. Clinical trials have shown cytisine is more effective than placebo and nicotine replacement therapy for achieving smoking abstinence for at least six months (De Santi et al., 2024; Lindson et al., 2023). The standard course of cytisine is 25 days, but when taken for 12 weeks it appears to achieve similar effectiveness to the 12-week course of varenicline, currently considered the most effective sole smoking cessation pharmacotherapy (Courtney et al., 2021; Lindson et al., 2023). Studies have also reported that cytisine is associated with fewer adverse events than varenicline (e.g., lower rates of nausea, abnormal dreams (Courtney et al., 2021)), which may make it a more acceptable option for those concerned about side effects (Courtney et al., 2021; Lindson et al., 2023).

Despite its demonstrated effectiveness and superior side-effect profile (compared with other effective medications such as varenicline), cytisine uptake in the UK since its introduction has been very low. Since 2024 only 0.2% of adults making a quit attempt reported using cytisine (Buss et al., 2025), and National Health Service (NHS) data indicate that only 0.7% of people accessing Stop Smoking Services (SSS) in England were prescribed cytisine since its introduction (NHS, 2025). The reasons for this low uptake are currently unclear, but it may be due to two key factors; healthcare professionals may not feel confident in prescribing cytisine as a relatively new smoking cessation medication in the UK, while people who smoke may be unaware of the medication, uncertain about its effectiveness, concerned about its safety, or reluctant to use a new smoking cessation medication. Although prescribing decisions are ultimately made by healthcare professionals, awareness and perceptions among people who smoke are also important because they can influence treatment preferences, willingness to use smoking cessation medications, and engagement with smoking cessation support. Consequently, this study focuses on understanding awareness, use and interest in future use of cysisine among people who smoke, as well as factors influencing this interest, to help identify barriers to uptake and inform educational and promotional strategies to maximise the public health impact of cytisine within UK smoking cessation support.

This study combined quantitative and qualitative data to provide a comprehensive understanding of awareness and use of cytisine among people who smoked in the past year, and interest in its use among people who currently smoke. The study also explored socio-demographic and smoking-related characteristics associated with awareness and interest in cytisine use and examined perceived barriers and facilitators to future cytisine use. Specifically, using a self-selected convenience adult sample in the UK, this study aimed to:

1. Examine awareness and use of cytisine among people who smoked in the past year, and interest in using cytisine among people who currently smoke.
2. Examine socio-demographic and smoking-related characteristics associated with awareness of cytisine among people who smoked in the past year, and interest in cytisine use among people who currently smoke.
3. Assess factors that may encourage cytisine use among people who currently smoke.

## 2 Methods

### 2.1 Study design

This study used an explanatory sequential mixed-methods design (Creswell & Clark, 2017). It consisted of an online survey followed by online semi-structured interviews with a sub-sample of the online survey participants. The interviews aimed to help explain the quantitative findings and generate greater insight into awareness of cytisine, interest in cytisine use and factors that may encourage use. Data collected between April–July 2025. The study was not pre-registered and should be regarded as exploratory. Accordingly, no formal sample size calculation was conducted, however we aimed to recruit approximately 200 responders, which allows estimation of prevalence outcomes with a margin of error of approximately ±7% at the 95% confidence level (assuming proportions around 50%), providing reasonably precise estimates of awareness and interest in cytisine use. Ethical approval was obtained from the University College London (UCL) Research Ethics Committee (0725).

### 2.2 Participants

Participants were eligible if aged ≥18, living in the UK, and having smoked in the past-year (currently smoking or quit within the past year). We included anyone who had smoked, even if quit, in the past year because cytisine has been available in the UK since January-2024 (NHS, 2024). Recruitment was through social media, flyers and Prolific (www.prolific.co). Interview participants were drawn from survey respondents who currently smoke, had not previously used cytisine and provided an email address.

### 2.3 Materials & procedure

A 10-min online survey was administered using REDCap (Harris et al., 2009; Harris et al., 2019). Participants provided socio-demographic and smoking-related characteristics and answered questions regarding cytisine. Upon completion, participants could enter a draw for a £25 voucher or receive £1 via Prolific. Although no specific bot-detection procedures were implemented, Prolific internal checks to ensure participant quality, and the modest incentive was unlikely to encourage fraudulent responses. Participants who expressed interest in participating in the interviews and provided their email address were contacted for interview. Interviews took place online, lasted ∼30 min, were audio-recorded and participants could enter a £50 voucher draw.

### 2.4 Measures

Survey measures were self-reported and have been used or formulated based on relevant questions of the Smoking Toolkit Study (Fidler et al., 2011). Original questions and response options are presented in supplementary Table 1.

#### 2.4.1 Smoking status

Participants reported daily/non-daily cigarette smoking, use of other tobacco products, recent quitting, and never-smoking. Respondents who currently smoked or had quit in the past year were included in the study. Those who had quit more than a year ago or had never smoked were excluded.

#### 2.4.2 Outcomes

Awareness of cytisine was assessed by asking whether participants were aware of cytisine, along with a brief description of cytisine. Responses were dichotomised as aware versus not aware (including “don't know”).

Use of cytisine among those who reported smoking in the last year and who made a serious quit attempt in that time was assessed by asking first participants which stop-smoking aids they had tried. Those who selected cytisine were then asked whether it had been offered by a general practitioner (GP; i.e., family doctor) or other healthcare professional. Participants who reported being offered cytisine were subsequently asked what they did with the offer. Participants who accepted the offer were classified as having used cytisine, and all others were classified as not used.

Interest in future cytisine use among those who currently smoke was assessed by asking how likely they would be to request cytisine from a GP or other healthcare professional in a future quit attempt. Those indicating that they were very/fairly likely to do so were classified as interested, and all others as not interested.

Factors associated with interest in using cytisine were assessed by asking participants which factors would encourage them to try cytisine as a smoking cessation medication. Participants could select multiple factors, and the full list of options is presented in Table 2.

#### 2.4.3 Covariates

Sociodemographic variables included age (continuous), sex (male/female), occupation (manual/other), post-16 qualification (education after the age of 16 years (yes/no)), ethnicity (white/other).

Smoking-related characteristics included number of cigarettes smoked/day, past-year quit attempts, use of support for smoking cessation in the most recent quit attempt (evidence-based including use any of face-to-face behavioural support, prescription medication, e-cigarettes, or over-the-counter nicotine replacement therapy)/all other (including none)), and among those who currently smoke we assessed motivation to quit as measured by the motivation to stop scale (Kotz et al., 2013) (high/low).

### 2.5 Interview schedule

The interview topic guide (supplementary Table 2) was structured around the COM-B model (Michie et al., 2011), which has three component areas: capability (psychological and physical), opportunity (social and physical) and motivation (reflective and automatic) and in varying combinations are considered essential to enable any behaviour to take place or be modified (Michie et al., 2011). COM-B is widely used to inform intervention development (Michie et al., 2011). The interview schedule contained 11 questions with 1–3 optional probe questions to help clarify responses, draw out examples, and encourage greater discussion. At the beginning of each interview, participants received a standardised introduction to cytisine. The interview topic guide was developed by JL and then discussed among the research team (JL, DK, MD).

### 2.6 Analysis

In line with the explanatory sequential mixed methods design, the two connected but different strands of data were analysed separately, and the findings are presented sequentially (Creswell & Clark, 2017). The findings from both strands were combined at an interpretative level to generate key conclusions.

Survey data were analysed using SPSS (version 28). There was no missing data. Proportions of participants who reported awareness, use and interest in future cytisine use were calculated. Descriptive statistics summarised sociodemographic, smoking-related characteristics, and factors encouraging future cytisine use.

For all those who reported smoking in the past year, unadjusted and adjusted logistic regression models examined associations between awareness and sociodemographic and smoking-related characteristics. Similarly, among those who currently smoke, unadjusted and adjusted logistic regression models examined the associations between interest in future cytisine use and sociodemographic and smoking-related characteristics. For all models, odds ratios (OR), 95% confidence intervals (CI), proportions, and *p*-values were reported, with statistical significance set at *p* < 0.05.

All interviews were transcribed verbatim, anonymised, and thematically analysed using a deductive, codebook based approach (Braun & Clarke, 2021) informed by the COM-B model (Michie et al., 2011) and coded using NVivo12 Plus (Jackson & Bazeley, 2019; Lumivero, 2018). Emerging themes were organised using the COM-B model. This framework provided a structured approach to relate the identified themes to participants' cytisine awareness and potential factors influencing future use. Participants sometimes expressed divergent or even opposing views on the same topic, and we aimed to represent this diversity within the themes. However, certain codes were excluded when they were too vague or lacked sufficient context to be meaningfully interpreted. This approach balanced the intention to capture a wide range of perspectives with the need for clarity and rigour in decisions about code inclusion. Three members of the research team (JL, MD, DK) each independently coded three interviews. Then they discussed it in the team, and the remaining interviews were coded by JL. The full codebook with the themes were shared with the research team (JL, MD, DK) and discussed and refined.

### 2.7 Researcher characteristics and reflexivity

The primary researchers (JL and MD) involved in data collection and analysis were Health Psychology Master's students at the time of the study. They were supervised by DK, a researcher at UCL Tobacco and Alcohol research group, whose research focuses on population and individual-level approaches to smoking cessation.

## 3 Results

### 3.1 Survey

A total of 193 people who reported smoking in the past year (mean age [Standard Deviation] = 37.66 [13.39]) completed the survey. Samples' sociodemographic and smoking-related characteristics are presented in Table 1.

**Table 1:** Sociodemographic and smoking-related of past-year smokers (*N* = 193).

**Sociodemographic characteristics**	
**Age, mean (SD)**	37.66 (13.39)
**Gender, n (%)**	
Male	104 (53.88)
Female	89 (46.12)
**Occupation, n (%)**	
Other	124 (64.24)
Manual	69 (35.76)
**Post-16 qualification, n (%)**	
Yes	164 (84.97)
No	29 (15.03)
**Ethnicity n (%)**	
White	126 (65.28)
Other	67 (34.72)
**Smoking-related characteristics**	
**Cigarettes per day, mean (SD)**	9.20 (5.12)
**Serious quit attempts in the last 12 months n (%)**	
No attempt	52 (26.94)
At least one attempt	141 (73.06)
Use of evidence-based support for smoking cessation in the most recent quit attempt, n (%)*	
Yes	127 (90.07)
No	14 (9.93)
Time spent smoking (years), mean (SD)**	16.28 (12.66)
Motivation to quit, n (%)**	
High	51 (30.18)
Low	118 (69.82)

* Among those who made at least one quit attempt in the last 12 months (n = 141), **among current smokers (n = 169), SD=Standard Deviation. For occupation ‘other’ includes: 72 (37.31%) non-manual, 25 (12.95%) student, 27 (13.98%) retired/unemployed). For ethnicity ‘other’ includes: 24 (12,43%) Asian, 23 (11.92%) Black, 16 (8.29%) Mixed, 4 (2.08%) other.

Forty-three (22.28%) people who smoked in the past year reported awareness of cytisine. Among those who made at least one serious quit attempt in the past year (*n* = 141), six (4.26%) reported cytisine use and all of them had been offered cytisine by their GP or other healthcare professional.

Among those currently smoking (*n* = 169), the most reported factors encouraging cytsine use in future quit attempts were: confidence that it would help with smoking cessation (52.66%), confidence that it is safe to use (46.74%) and availability of clinical evidence demonstrating that cytisine is effective (45.56%) (Table 2). Additionally, 121 (71.60%) expressed interest in future cytisine use.

**Table 2:** Factors encouraging cytisine use in future quit attempts (*n* = 169).

**Factors encouraging cytisine use among current smokers**	**n (%)**
Confident it would help	89 (52.66)
Confident that it is safe to use	79 (46.75)
Motivated to stop smoking	61 (36.09)
Last resort (if nothing else worked)	28 (16.57)
Understand how to use the medication	66 (39.05)
Understand the medication itself	70 (41.42)
See clinical evidence of whether it works or not	77 (45.56)
See or hear about someone else having used it successfully	58 (34.32)
Confident it would be easy to get a prescription	56 (33.14)
Other	3 (1.77)
Don't know/none of these	6 (3.55)

Awareness of cytisine was associated with manual occupation (adjusted odds ratio (aOR) = 6.25, 95%CI 2.78, 14.29), past-year serious quit attempts (aOR) = 3.21, 95%CI 1.09, 9.51) and smoking fewer cigarettes /day (aOR = 0.89, 95%CI 0.80, 0.98) (Table 3). Interest in future cytisine use was associated with past-year quit attempts (aOR = 3.88, 95%CI 1.70, 8.88) and past use of evidence-based smoking cessation support (aOR = 13.21, 95%CI 1.89, 92.2) (Table 3). Unadjusted analyses are presented in supplementary Table 3.

**Table 3:** Adjusted association between cytisine awareness and sociodemographic and smoking-related characteristics among past-year smokers (n = 193) and between interest in using cytisine and sociodemographic and smoking-related characteristics among current smokers who were interest in using cytisine in future quit attempts (*n* = 121).

	**n Cytisine awareness (%)**	**aOR** **(95% CI)**	**p-value**	**n Interest in using cytisine (%)**	**aOR** **(95% CI)**	**p-value**
**Sex**						
Male	23 (22.12)	(ref)		72 (78.3)	(ref)	
Female	20 (22.47)	1.24 (0.55, 2.77)	0.602	49 (63.6)	0.49 (0.22, 1.07)	0.072
**Occupation**						
Other	13 (10.48)	(ref)		71 (67.0)	(ref)	
Manual	30 (43.48)	6.25 (2.78, 14.29)	**<0.001**	50 (79.4)	1.14 (0.48, 2.63)	0.773
**Post-16 qualification**						
Yes	37 (22.56)	(ref)		102 (70.8)	(ref)	
No	6 (20.69)	1.71 (0.52, 5.61)	0.374	19 (76.0)	0.75 (0.23, 2.44)	0.628
**Ethnicity**						
White	23 (18.25)	(ref)		72 (68.6)	(ref)	
Other	20 (29.85)	1.67 (0.71, 4.01)	0.235	49 (76.6)	1.99 (0.81, 4.92)	0.136
**Serious quit attempt in the past year**						
None	5 (9.62)	(ref)		26 (50.0)	(ref)	
At least one	38 (26.95)	3.21 (1.09, 9.51)	**0.035**	95 (81.2)	3.88 (1.70, 8.88)	**0.001**
**Use of evidence-based support for smoking cessation in most recent quit attempt**⁎						
No	1 (7.14)	(ref)		5 (41.7)	(ref)	
Yes	27 (29.12)	9.66 (0.97, 93.57)	0.060	90 (85.7)	13.21 (1.89, 92.2)	**0.009**
**Motivation to quit**						
High	**–**	**–**		42 (82.4)	(ref)	
Low	**–**	**–**	**–**	79 (66.9)	0.59 (0.24, 1.47)	0.258
	**M (SD)**	**aOR** **(95% CI)**	**p-value**	**M (SD)**	**aOR** **(95% CI)**	**p-value**
**Age**	33.86 (11.94)	1.00 (0.96, 1.04)	0.884	38.61 (12.73)	1.06 (0.99, 1.14)	0.093
**Cigarettes/day**	7.72 (4.72)	0.89 (0.80, 0.98)	**0.019**	9.70 (5.01)	0.97 (0.87–1.09)	0.656
**Time spent smoking (years)**	–	–	**–**	16.92 (12.63)	0.97 (0.90, 1.04)	0.383

⁎ Among those who made at least one quit attempt in the last 12 months (n = 141). Adjusted for sociodemographic and smoking-related characteristics. aOR = adjusted odds ratio, CI=Confidence Intervals, ref. = reference category, M = mean, SD = standard deviation.

### 3.2 Interviews

**Fig. 1:**
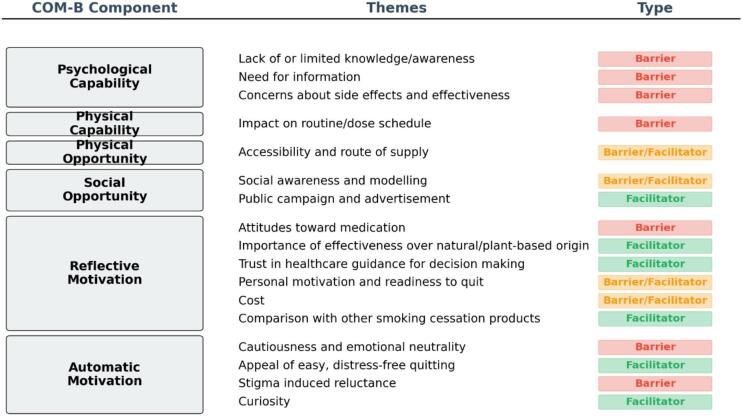
Qualitative analysis: Themes mapped to COM-B model.

Sixty-five participants who currently smoked agreed to be contacted for the interview, 25 chose an interview date, nine did not attend, and 16 completed the interview (mean age [Standard Deviation] = 34.44 [12.85]). The sample included 10 females, seven participants were White, 12 reported high motivation to quit and six had made at least one past-year quit attempt (supplementary Table 4 describes participants' characteristics). Thematic saturation was achieved, with no new themes emerging in the final five interviews analysed. We identified 17 themes in total reflecting a range of barriers and facilitators influencing interest in future cytisine use (Fig. 1: themes mapped to COM-B; Table 4: themes and quotes).

**Table 4:** Qualitative analysis results.

**COM-B**	**Key Themes**	**Barrier/ Facilitator**	**Quote**
Psychological Capability	Lack of or limited knowledge/ awareness	Barrier	P3: “No, I never heard of it… I've not seen it or heard of it, whether it be through advertisements, posters in the tube, or the radio or TV.” P12: “I've heard a little bit about it, but not much… at a giving up smoking clinic… I just got a small leaflet about it that sort of said I could have it from the GP, but I didn't do any research into it...”
	Need for information	Barrier	P1: “I'd want a little bit more about kind of the dose…how long you have to take it for…how it interacts with other medication.” P2: “I need to hear more about, for example, what the participants in a clinical trial felt about this medication, what is the percentage of efficacy?” P3: “Maybe like statistics or…just some more like information about how it works and if it's worked for other people.” P7: “…since I never heard of it, I'm thinking of what it is, would that be effective or not?” P13: “I would like to know what are the components… so how does it work? What does it do? Does it affect anything else in my body?”
	Concerns about side effects and effectiveness	Barrier	P1: “Perhaps…how it would make me feel, if there would be any side effects.” P2: “I would be very cautious about them because they are kind of interacting with our neuro systems, especially for nicotine that makes some of us like more active than usual. So if you said [it] will cause some side effects like drowsiness, like brain fog? Then it's just not. It's just not necessary. It's like, opposite of what I wanted.” P3: “I'd be fine to use it…just read a little bit about side effects, what they might be.” P5: “…the main like thought or question that comes to mind is… does the medicine itself become addictive is kind of what I would wonder or is it basically to transition off of nicotine and then you stop the medication.” P14: “I maybe want more fundamental information…what's the side effects? What's any kind of maybe negative effects to our body?” P16: “I actually did a look up because my [condition] medication… So I have to be sure that whatever else I take… I want to be able to check what the liver interactions are...”
Physical capability	Impact on routine/dose schedule	Barrier	P5: “…the mode of administration and things like practicalities as well would be a factor. If it's one of those pills that you have to take five times a day, I would probably be less likely to do it.” P13: “If it's something complicated, and I have to do something and remember something and be a chemist to… No, I'm not going to do that.”
Physical opportunity	Accessibility and route of supply	Barrier/ Facilitator	P1: “…if you're waiting for a GP appointment for two weeks, and then in those two weeks you're urged to…your desire to stop changes, then you're kind of back to square one and probably not going to the GP and getting the asking for the medication. I think it would be good to be over the counter because it would perhaps [be] more accessible for people, and people [would] be more likely to purchase it or use it [so] that they don't have to go to the GP and have an awkward conversation” P3: “I feel like I'd be less inclined to get a prescription from the GP because it just feels like more processed to go to the GP, get appointment and get the prescription rather than it being like just available over the counter that I can get.” P2: “If it is a prescription medication, then it would be, a lot less accessible, but if it is like those nicotine patches that you can get from Boots, then it's a lot more accessible to me.” P5: “…Rather than like fully pick it off the shelf and buy it, I think OTC but talking to a pharmacist.” P11: [I]’d probably be more likely to try that over the counter than something else.” P13: “If it can be through something like you get a birth control pill or like some other medication where you kind just get it quickly, and it's your pharmacy next to you and you just pick it up.”
Social opportunity	Social awareness and modelling	Barrier/ Facilitator	P1: “Mainly just sort of like vapes or gum…those kinds of tools are really well known. …the most important thing for me would be if I wanted to do it, as opposed to being influenced by other people…but it's encouraging and positive to me.” P3: “I don't know anyone who's using something to stop them smoking. Maybe if I knew someone who smoked a lot…they were really happy with what they used…then maybe yeah…I'd consider it. P8: “I think if someone I knew tried it and it worked for them, I'd be a lot more likely to also try it...”
	Public campaign and advertisement	Facilitator	P11: “…I know obviously it's prescriptions. I'm pretty sure there's probably some laws around that can't advertise it, but even just some leaflets available at the doctors or something like, ‘oh, you can ask the doctor about this kind of thing’.”
Reflective motivation	Attitudes toward medication	Barrier	P7: “I am not willing to use any medications for quit smoke…” P1: “…because it's a medication. It feels a lot more serious than the gums or vapes or the patches or whatever other patches…and because it's a prescription only, it does feel more kind of like serious.” P2: “…I'm not that easily getting myself into a situation where I have to rely on the medication to quit something.” P13: “Prescription is needed for things that do have some severe side effects or that can be abused… some medication that can actually harm me if I take too much or for too long.”
	Importance of effectiveness over natural/plant-based origin	Facilitator	P1: “As long as I know it would work; I wouldn't be as concerned about the plant-based natural…more concerned about the outcome.” P2: “I appreciate that it's plant-based, so it feels healthier…, but ultimately is its effectiveness.” P6: “it's an interesting concept because it's plant-based alternative…is it effective?”
	Trust in healthcare guidance for decision making	Facilitator	P11: “I think I'd be more confident if… would be better if it was like something that the GP was like, ‘oh, we think this would be the best medication for you’, I'd kind of trust them… because obviously I feel like they would know more about it than I would.” P10: “…you always think that the stuff you can just buy is less potent than, like if you want proper painkillers, you get the doctor prescribed you, paracetamol sometimes doesn't work.” P15: “…apart from doing some research myself, I may still follow my doctor's advice. If they give me some options and if this product is in one of those options, then I may do.” P12: “…there is that layer of trust involved…the NHS does its research, so you know you're not going to be taking anything harmful.”
	Personal motivation and readiness to quit	Barrier/ Facilitator	P2: “…because smoking for me, I not, I'm not addicted…smoking was never really a problem for me…and if I want, I can stop.” P11: “I think it's just the will. I know from starting previously, you have to want to stop, otherwise it's never going to work.”
	Cost	Barrier/ Facilitator	P1: “I think the cost…if it works then it's a short-term cost…probably save money in the long term.” P3: “It depends on the proportion…if it's really expensive but really effective, I might choose a cheaper option that's still almost just as effective. P9: “…the way I think about it, smoking is expensive habit. So, any cost on the drug that is within the cost of a packet of cigarettes is absolutely reasonable.” P12: “A prescription is a fixed rate, and you can get other…giving up smoking aids, Nicorette and all those nicotine, all those brands on prescription and over the counter. But they're invariably three times the price, which is, you know, is important.” P12: “I would rather spend more money on something that worked than less money on something that isn't going to work.”
	Comparison with other smoking cessation products	Facilitator	P6: “I think [I] will need to know that there are lesser side effects, or there was more chance of you stop smoking to the other products that are on the market.” P10: “… some of the other products on the market, you can't take them indoors and stuff…if it's like a tablet form or…that would obviously make a difference.”
Automatic motivation	Cautiousness and emotional neutrality	Barrier	P1: “I've not got any sort of strong reaction to it to it either way, I guess.” P13: “My initial thoughts are same as any new medication, it sounds…interesting… [but also] I'm very suspicious of new things…”
	Appeal of easy, distress-free quitting	Facilitator	P2: “…if they want to quit smoking, like really badly, then if you tell them this medication can help them just go cold turkey on smoking without any distress, it's very attractive. It must be very attractive to many people.” P3: “…smooth quitting process and just overall satisfaction with what they've used.”
	Stigma induced reluctance	Barrier	P1: “People just don't like to admit that they couldn't quit if they wanted to…using willpower or using the odd bit of gum…that would be a big…thing to acknowledge and admit to themselves that they needed something to help them stop.” P7: “… don't really like admitting they can't just stop…, even if they've tried”.
	Curiosity	Facilitator	P9: “…because I haven't tried it. That's one thing that will make me want to try it out because it's a new thing. I haven't done it before, so I don't know if it works or not. And that curiosity.” P15: “…maybe I will give it a got…just to see if it works.”

*Psychological Capability “I've heard a little bit about it, but not much…at a giving up smoking clinic*” [Participant_12: Female, 57].

Lack of or limited prior knowledge/awareness (Barrier): Participants mentioned that they had never heard of cytisine before this study, except for two who had previously come across it through leaflets or in journal articles.

Need for more information (Barrier): Participants expressed a desire for further information about cytisine, particularly regarding its dosage and mechanism of action.

Concerns about side effects and effectiveness (Barrier): Concerns were raised about cytisine's potential side-effects, real-world effectiveness, and its potential risk of addiction due to its similar structure to nicotine.

*Physical Capability: “If it's one of those pills that you have to take five times a day, I would probably be less likely to do it”* [Participant_5: Female, 22].

Impact on routine/dose schedule (Barrier): It was suggested that the complexity and/or high frequency of cytisine use might be difficult to integrate into daily life, which could therefore be a potential barrier to cytisine use.

*Physical Opportunity: “If it is a prescription medication, then it would be, a lot less accessible”* [Participant_2: Female, 23].

Accessibility and route of supply (Barrier/Facilitator): It was noted that the requirement for a prescription could be interpreted as a practical obstacle, as it increases the complexity of obtaining the medication and limits the immediacy of access during moments of strong smoking urges. While participants felt that, if it was available over the counter, it could improve accessibility and convenience.

*Social Opportunity: “I think if someone I knew tried it and it worked for them, I'd be a lot more likely to also try it...”* [Participant_8: Female, 20].

Social awareness and modelling (Barrier/Facilitator): Participants highlighted the lack of peer discussion or role models for cytisine use, especially compared with more visible and popular smoking cessation aids. However, they noted that hearing about positive experiences from peers who successfully used cytisine could increase their trust and willingness to try it.

Public campaign and advertisement (Facilitator): It was suggested that increased visibility through public health campaigns or accessible informational materials could raise awareness and prompt questions about cytisine to healthcare professionals, potentially facilitating cytisine use.

*Reflective Motivation: “I'm not that easily getting myself into a situation where I have to rely on the medication to quit.”* [Participant_2: Female, 23].

Attitudes toward medication (Barrier): Participants expressed general reluctance toward using medication to support a quit attempt. The prescription-only status of cytisine made it feel more “serious” or potentially risky compared with over-the-counter cessation aids. Some associated prescribed medications with stronger pharmacological effects, possible dependence, or long-term harm, contributing to hesitancy toward cytisine use.

Trust in healthcare guidance for decision-making (Facilitator): It was highlighted that guidance from healthcare professionals played a crucial role in considering cytisine use. Participants felt reassured that recommendations from trusted sources indicated safety and appropriateness. While this trust generally facilitated interest in cytisine, some reported occasional reservations due to perceived gaps in health professionals' knowledge about smoking cessation medications.

Importance of effectiveness over natural/plant-based origin (Facilitator): The plant-based origin of cytisine was viewed as a positive feature that contributed to perceptions of safety. However, participants prioritised the drug's effectiveness over its natural origin.

Personal motivation and readiness to quit (Barrier/Facilitator): Personal motivation level and readiness to quit smoking were fundamental to considering any cessation aid. This factor functioned as a facilitator for those with high readiness and a barrier for those who felt less addicted or unprepared to quit.

Cost (Barrier/Facilitator): Cost was an important factor for participants when considering smoking cessation aids, although treatment effectiveness often outweighed price in decision-making.

Comparison with other smoking cessation products (Barrier/facilitator): Participants indicated that their decision would be influenced by direct comparisons between cytisine and other available smoking cessation aids.

*Automatic Motivation: “My initial thoughts are same as any new medication, it sounds…interesting… [but also] I'm very suspicious of new things…”* [Participant_13: Male, 35].

Cautiousness and emotional neutrality (Barrier): A generally cautious or neutral stance toward new medications such as cytisine was reported. Some participants expressed no strong initial reaction to cytisine, while others described inherent suspicion toward unfamiliar pharmaceutical treatments, representing a potential barrier to engagement.

Appeal of easy, distress-free quitting (Facilitator): The prospect of a smoother and more manageable quitting process was highly appealing for some participants, and it could act as a facilitator when considering whether to use cytisine.

Stigma-induced reluctance (Barrier): Some participants described an internalised stigma around using medication for smoking cessation, viewing it as an admission that willpower alone was insufficient.

Curiosity (Facilitator): The novelty of cytisine served as a facilitator for participants. They expressed willingness to try cytisine simply because it was new to them.

## 4 Discussion

This mixed-methods study found low awareness and use of cytisine among people who smoked in the past year, with fewer than a quarter aware of cytisine and only a small number reporting use. In contrast, interest in future cytisine use among those who currently smoke was high, with nearly three-quarters reporting interest in future cytisine use. Awareness and interest in future use were higher among those with a quit attempt in the past year, while awareness was also positively associated with smoking fewer cigarettes per day and future cytisine use with past use of evidence-based smoking cessation support.

The low levels of cytisine use observed in this study, while higher than previous national estimates, are broadly consistent with trends reported elsewhere (Buss et al., 2025; NHS, 2025). These patterns suggest that cytisine remains largely unfamiliar to people who smoke despite its established efficacy in helping people to quit (De Santi et al., 2024; Lindson et al., 2023). In contrast, our results indicate high interest in future cytisine use, suggesting that uptake could increase if more people were aware of its availability, proven effectiveness, and safety. This finding is broadly consistent with qualitative research exploring interest in cytisine use among indigenous people who smoke in New Zealand, which highlighted limited prior knowledge of cytisine but high interest in future use (Thompson-Evans et al., 2011).

Survey results showed that interest in future cytisine use was influenced by confidence in its effectiveness and safety, the availability of clinical evidence, and the clarity of instructions. These findings highlight the importance of informational and practical factors in supporting uptake. Similar patterns have been observed for other smoking cessation medications, where greater knowledge of safety and efficacy is associated with increased use, while lower uptake is often linked to factors such as cost, access, limited awareness, misperceptions about effectiveness or safety, and concerns about addiction (Bansal et al., 2004; Smith et al., 2015).

The qualitative findings closely reflected the survey results and provided more detailed insight into barriers and facilitators shaping interest in future cytisine use. Participants emphasised the need for more information about cytisine, especially its mechanism of action and real-world effectiveness. Consistent with previous research on other smoking cessation medications (Smith et al., 2015), participants raised concerns that could reduce willingness to consider future cytisine use such as side effects, perceived addiction risk due to similarity to nicotine, and uncertainty about cytisine's effectiveness. Individual attitudes toward pharmacotherapy also shaped interest in future cytisine use. Some participants expressed caution or stigma-induced reluctance toward using smoking cessation medication, while others were curious about cytisine and its potential to support an easier, less distressing quit attempt. Although participants viewed cytisine's plant-based origin positively, perceived effectiveness remained the priority, consistent with evidence that perceived effectiveness strongly influences intention to use smoking cessation pharmacotherapy (Vogt et al., 2008).

Practical and contextual factors further shaped interest in future cytisine use. Participants described the frequency and complexity of cytisine dosing as difficult to integrate into daily life, consistent with prior research on cytisine (Thompson-Evans et al., 2011). Cytisine's accessibility and route of supply were perceived as both a barrier and a facilitator; prescription-only access was seen as limiting convenience and immediacy for some, while for others it increased confidence in cytisine through healthcare endorsement of safety. Limited awareness and modelling of cytisine use, especially compared with established cessation aids (e.g., e-cigarettes), also constrained interest in future cytisine use. In contrast, positive peer experiences and increased visibility through public campaigns could build trust in cytisine and prompt discussions with healthcare professionals, reflecting evidence from research on e-cigarettes (Jackson, Squires, et al., 2025).

Overall, these findings highlight how informational gaps, practical constraints, social visibility, and individual attitudes interact to influence interest in cytisine use, complementing and extending the survey results by identifying specific, modifiable factors that may shape future uptake. These findings can inform future research and interventions aimed at increasing awareness and understanding of cytisine, particularly through healthcare professionals and public health messaging. Improving information on effectiveness, safety, and use, alongside greater visibility within smoking cessation services, may support informed decision-making and uptake. From a policy perspective, integrating cytisine more clearly into smoking cessation pathways could help translate interest into use.

This study has several strengths, including the use of a mixed methods design that enabled both quantitative analysis and qualitative explanation of the factors shaping future cytisine use, and being one of the first studies to examine awareness, use, and interest in cytisine in a UK context. However, several limitations must be acknowledged. First, the survey relied on a small convenience sample, which limits generalisability, may overrepresent individuals interested in smoking research, and introduces potential self-selection bias as those with greater interest in smoking cessation or novel treatments may have been more likely to take part. Second, as the survey was conducted online, certain groups such as older adults or those with lower digital literacy, may be underrepresented, and although the compensation was minimal, there remains potential risk of automated or fraudulent responses (Goodrich et al., 2023). Third, the survey relied on self-reported measures, which may be subject to recall bias, particularly for past quit attempts and prior use of smoking cessation medication, potentially leading to under- or over-reporting of these behaviors. Fourth, the low response rate for the qualitative interviews, potentially due to the greater time and commitment required compared with completing the online survey, may have introduced selection bias. As participants who took part were more likely to report high motivation to quit compared with the wider survey sample. This may have influenced the findings by over-representing individuals more receptive to using a novel smoking cessation medication such as cytisine. Fifth, qualitative findings, while rich and insightful, represent the views of a small group and may not reflect the broader population. Lastly, this study only assessed interest in using cytisine, which may not translate into real-world uptake.

## 5 Conclusion

To conclude, this mixed-methods study suggests that awareness and use of cytisine among those who smoked in the past year were low. In contrast, interest in future use among those who currently smoke was high. Interest in future cytisine use is influenced by informational, practical, social, and motivational factors, suggesting that increased knowledge, visibility, and reassurance about cytisine's effectiveness and safety could encourage smokers to consider it in future quit attempts.

## CRediT authorship contribution statement

**Megan Devereux:** Writing – original draft, Formal analysis, Data curation, Conceptualization. **Jie Lyu:** Writing – original draft, Formal analysis, Data curation, Conceptualization. **Vera Helen Buss:** Writing – review & editing. **Sharon Cox:** Writing – review & editing. **Lion Shahab:** Writing – review & editing. **Dimitra Kale:** Writing – review & editing, Supervision, Resources, Methodology, Conceptualization.

## Funding

This research received no specific funding. DK receives salary support from 10.13039/501100000289Cancer Research UK (PRCRPG-Nov21\100002). VHB and SC receives salary support from the Behavioural Research UK Leadership Hub, which is supported by the UK Economic and Social Research Council (ES/Y001044/1), and 10.13039/501100000289Cancer Research UK (PRCRPG-Nov21\100002).

For the purpose of Open Access, the author has applied a CC BY public copyright licence to any Author Accepted Manuscript version arising from this submission.

## Declaration of competing interest

The authors declare that they have no known competing financial interests or personal relationships that could have appeared to influence the work reported in this paper.

## Data Availability

Data will be made available on request.
