# Supplementary material for: Awareness, use and future interest in cytisine for smoking cessation in adults who smoke or have quit within the past year in the UK: A mixed-methods study
Source: Addict Behav Rep. 2026 Jun 30;24:100726. doi: 10.1016/j.abrep.2026.100726 (PMC13351444; doi:10.1016/j.abrep.2026.100726)
Supplement: Supplementary file 1 — Supplementary material [file mmc1.docx]

Supplementary Table 1. Original survey questions and answer options. Questions are derived from Smoking Toolkit Study.

| Measure | Original Question | Answer Options |
| --- | --- | --- |
| Smoking status | “Which of the following best applies to you? Please note we are referring to cigarettes and other kinds of tobacco that you set light to and NOT electronic or ‘heat-not-burn’ cigarettes.” | a. I smoke cigarettes (including hand-rolled) every day.  b. I smoke cigarettes (including hand-rolled), but not every day. c. I do not smoke cigarettes at all, but I do smoke tobacco of some kind (e.g., pipe, cigar or shisha).  d. I have stopped smoking completely in the last year.  e. I stopped smoking completely more than a year ago.  f. I have never been a smoker (i.e., smoked for a year or more). |
| Awareness of cytisine | Are you aware of cytisine? Cytisine is a prescription-only medicine to help you stop smoking, available in the UK since 2024. It does not contain nicotine but mimics its effects, ‘tricking’ your brain into thinking you have had a cigarette. It is plant-based and helps reduce the urge to smoke, minimises withdrawal symptoms, and relieves cravings. | a. Yes  b. No  c. Don’t know |
| Use of cytisine | 1) Which, if any, of the following did you try to help you stop smoking during the most recent serious quit attempt?  And if selected cytisine, they were asked  2) Were you offered cytisine by a GP, or other healthcare professionals?  3) Which if any best describes what you did with the offer of cytisine? | 2a. Yes  2b. No  3a. Accepted the offer  3b. Did not accept  3c. Still deciding  3d. Don’t know |
| Interest in future use | How likely are you to ask your GP, or other healthcare professional, to prescribe you cytisine in the future? | a. Very likely  b. Fairly likely  c. Not very likely  d. Not at all likely  e. Don’t know |
| Factors associated with interest | Which of the following, if any, would encourage you to try cytisine as a stop smoking medication? | a. Confident it would help.  b. Confident that it is safe to use.  c. Motivated to stop smoking.  d. Last resort (if nothing else worked).  e. Understand how to use the medication.  f. Understand the medication itself.  g. See clinical evidence of whether it works or not.  h. See or hear about someone else having used it successfully.  i. Confident it would be easy to get a prescription.  j. Other.  k. Don’t know/none of these. |
| Age | Please provide your age in years. | 18-99 |
| Sex | Which of the following describes how you feel about yourself? | a. Male  b. Female  c. In another way  d. Prefer not to say |
| Occupation | Which of the following best describes your job? | a. Manual  b. Non manual  c. Student  d. Other (e.g., retired, unemployed) |
| Post-16 qualification | Do you have any post-16 qualifications (e.g., T-levels, A levels, University degree)? | a. Yes  b. No |
| Ethnicity | What is your ethnicity? | a. Any Asian or Asian British background  b. Any Black, Black British, Caribbean or African background.  c. Any White background.  d. Mixed or multiple ethnic background (e.g., White and Black African or White and Asian).  e. Other ethnic group (e.g., Arab). |
| Cigarettes smoked/day | How many cigarettes do you usually smoke a day/ on the days when you smoke? | 1-99 |
| Past-year quit attempts | How many serious attempts to stop smoking have you made in the last 12 months? By serious attempt, I mean you decided that you would try to make sure you never smoke again. Please include any attempt that you are currently making and please include any successful attempt made within the last year. | 0-99 |
| Use of support for smoking cessation | Which if any of the following did you try to help you stop smoking during the most recent serious quit attempts? | 1.) Nicotine replacement product (e.g., patches/gum/inhaler) without a prescription. 2.) Nicotine replacement product on prescription or given to you by a health professional.  3.) Zyban (bupropion).  4.) Champix (varenicline).  5.) Cytisine.  6.) Tobacco-free nicotine pouch/pod or 'white pouches' that you place on your gum (e.g. Zyn, On!, Nordic Spirit, Velo, Lyft, Skruf).  7.) Attended a Stop-Smoking group.  8.) Attended one or more Stop-Smoking one-to-one counselling/advice/support sessions.  9.) Phoned a smoking helpline.  10.) Visited NHS smokefree website.  11.) Electronic cigarette or vaping device.  12.) Used an application on a handheld computer (smartphone, tablet, PDA).  13.) Hypnotherapy.  14.) Acupuncture.  15.) Heat-not-burn (e.g., iQOS with HEETS, heatsticks.  16.) Juul.  17.) Book.  18.) Other.  19.) None of these.  Responses were dichotomised into ‘Evidence-based treatments’ (options 1-11) and ‘non-evidence-based treatments’ (options 12-19). Participants were able to select multiple options for this question. |
| Motivation to quit | Which of the following best describes you? | a) I REALLY want to stop smoking and intend to in the next month.  b) I REALLY want to stop smoking and intend to in the next 3 months.  c) I REALLY want to stop smoking, but I don't know when I will.  d) I want to stop smoking and hope to soon.  e) I want to stop smoking but haven't thought about when.  f) I think I should stop smoking but don't really want to.  g.) I don't want to stop smoking.  Responses were dichotomised into high (a–b) and low motivation (c–g and any response of “I don’t know”). |

Supplementary Table 2. Interview guide

| **SECTION 1: Capability**  Understanding participants’ knowledge, confidence, and psychological capacity to use cytisine. |
| --- |
| **Awareness and Knowledge of Cytisine** |
| 1.Have you ever heard about cytisine before? |
| If YES: Can you describe what you know about cytisine?  Probe: Where did you first hear about it?  Probe: How reliable do you think this information is?  Thank you for sharing. I understand you may already be familiar with cytisine, but I’d like to briefly go over some key information to make sure we’re on the same page. It is a plant-based medication, and it works by competing with nicotine and partly taking the place of nicotine binding-receptors in your brain, so you don’t feel the same cravings when you try to quit. Previous clinical trial has shown that it has less side effects than other stop smoking medication.  Are there any aspects of the information that you have about cytisine that you find confusing or unclear?  Probe: Would you feel confident using it based on your current knowledge? |
| If NO: No worries, I could provide you with some information about cytisine. It is a plant-based medication, and it works by competing with nicotine and partly taking the place of nicotine binding-receptors in your brain, so you don’t feel the same cravings when you try to quit. Previous clinical trial has shown that it has less side effects than other stop smoking medication.  Now that you’ve heard a brief introduction, what are your initial thoughts about it?  Probe: Would you feel confident using it based on this current information? |
| **Understanding of Usage** |
| 2.If you were to consider using cytisine, what kind of instructions or information would you need to feel confident about its use?  Probe: Would concerns about how to take it or potential side effects affect your decision?  Probe: Research suggests cytisine is one of the most effective stop-smoking medications, would that make you more likely to try it?  Probe: (As we mentioned earlier) Cytisine is plant-based and clinical trials suggest it has fewer side effects than other stop-smoking medications. Would these characteristics influence your willingness to try it? |
| **SECTION 2: Opportunity**  Exploring external or environmental factors that might support or hinder cytisine use. |
| **Accessibility and Availability** |
| 3. Do you think cytisine is easily accessible to you? Why or why not?  Probe: How would availability through GPs or pharmacies influence your decision? |
| 4.Currently, cytisine is only available under prescription in the UK. Do you think it should be available over the counter?  Probe: Do you think you’d be more willing to try it if you didn’t need a prescription? |
| **Social and Environmental Influences** |
| 5. Have you ever discussed cytisine or other smoking cessation aids with anyone?  Probe: Your GP, family, or friends? What were their opinions? How much do others' opinions influence your choices? |
| 6.Can you think of a time when someone’s success story with a smoking cessation aid influenced your behaviour or decision-making?  Probe: Would hearing similar stories about cytisine encourage you to try it? |
| **SECTION 3: Motivation**  Investigating participants’ reflective and automatic motivations. |
| **Interest in Using Cytisine** |
| 7.Based on what you know now, how likely are you to try cytisine to quit smoking?  Why?  Probe: What specific factors make you more or less likely to try it? |
| 8. When thinking about stop-smoking aids, is cost more important to you than effectiveness? |
| 9. (*Do not ask if participant states they never tried/considered any stop-smoking aids to quit smoking before).* How does cytisine compare to other smoking cessation aids you’ve considered or tried?  Probe: What makes it more appealing or less appealing in your view? |
| **Barriers and Facilitators** |
| 10. What do you see as the biggest barriers to trying cytisine?  Probe: Are these related to safety, effectiveness, supply (since it is available only under prescription) or something else? |
| 11. What might make you more confident or motivated to try cytisine?  Probe: Would clinical evidence, endorsements from healthcare providers, or personal recommendations help? |

Supplementary Table 3. Unadjusted association between cytisine awareness and sociodemographic and smoking-related characteristics among past-year smokers (n=193) and between interest in using cytisine and sociodemographic and smoking-related characteristics among current smokers who were interest in using cytisine in future quit attempts (n=121)

|  | **n Cytisine awareness (%)** | **OR**  **(95% CI)** | **p-value** | **n Interest in using cytisine (%)** | **OR**  **(95% CI)** | **p-value** |
| --- | --- | --- | --- | --- | --- | --- |
| **Sex** | | | | | | |
| Male | 23 (22.12) | (ref) |  | 72 (78.3) | (ref) |  |
| Female | 20 (22.47) | 1.02  (0.52, 2.02) | 0.953 | 49 (63.6) | 0.49  (0.25, 0.96) | **0.037** |
| **Occupation** | | | | | | |
| Other | 13 (10.48) | (ref) |  | 71 (67.0) | (ref) |  |
| Manual | 30 (43.48) | 6.67  (3.13, 14.23) | **<0.001** | 50 (79.4) | 1.89  (0.91, 4.00) | 0.087 |
| **Post-16 qualification** | | | | | | |
| Yes | 37 (22.56) | (ref) |  | 102 (70.8) | (ref) |  |
| No | 6 (20.69) | 0.90  (0.34, 2.36) | 0.823 | 19 (76.0) | 1.30  (0.49, 3.49) | 0.598 |
| **Ethnicity** | | | | | | |
| White | 23 (18.25) | (ref) |  | 72 (68.6) | (ref) |  |
| Other | 20 (29.85) | 1.91  (0.95, 3.81) | 0.068 | 49 (76.6) | 1.50  (0.74, 3.05) | 0.265 |
| **Serious quit attempt in the past year** | | | | | | |
| None | 5 (9.62) | (ref) |  | 26 (50.0) | (ref) |  |
| At least one | 38 (26.95) | 3.47  (1.28, 9.37) | **0.014** | 95 (81.2) | 4.32  (2.11, 8.82) | **<0.001** |
| **Use of evidence-based support for smoking cessation in most recent quit attempt*** | | | | | | |
| No | 1 (7.14) | (ref) |  | 5 (41.7) | (ref) |  |
| Yes | 27 (29.12) | 5.26  (0.68, 50.00) | 0.112 | 90 (85.7) | 8.40  (2.36, 29.95) | **0.001** |
| **Motivation to quit** | | | | | | |
| High | **-** | **-** | **-** | 42 (82.4) | (ref) |  |
| Low | **-** | **-** | **-** | 79 (66.9) | 0.43  (0.19, 0.98) | **0.045** |
|  | **M (SD)** | **OR**  **(95% CI)** | **p-value** | **M (SD)** | **OR**  **(95% CI)** | **p-value** |
| **Age** | 33.86 (11.94) | 0.97  (0.94, 0.99) | **0.037** | 38.61 (12.73) | 1.03  (1.00, 1.06) | **0.046** |
| **Cigarettes/day** | 7.72 (4.72) | 0.92  (0.86, 0.99) | **0.021** | 9.70 (5.01) | 1.08  (1.01, 1.15) | **0.033** |
| **Time spent smoking (years)** | - | - | **-** | 16.92 (12.63) | 1.02  (0.99, 1.04) | 0.296 |

* Among those who made at least one quit attempt in the last 12 months (n=141). OR=odds ratio, CI=Confidence Intervals, ref=reference category, M=mean, SD=standard deviation.

Supplementary Table 4. Characteristics of interview participants

| **Participant** | **Age**  **(Years)** | **Gender** | **Ethnicity** | **Cigarettes/day** | **Motivation to quit** | **Past-year quit attempts** |
| --- | --- | --- | --- | --- | --- | --- |
| 1 | 35 | Female | White | 5 | High | 0 |
| 2 | 23 | Female | Other | 2 | Low | 0 |
| 3 | 24 | Female | Other | 2 | High | 0 |
| 4 | 25 | Female | Other | 3 | High | 0 |
| 5 | 22 | Female | White | 3 | High | 3 |
| 6 | 45 | Male | Other | 10 | High | 8 |
| 7 | 24 | Male | Other | 12 | High | 0 |
| 8 | 20 | Female | Other | 4 | Low | 0 |
| 9 | 50 | Male | Other | 15+ | High | 0 |
| 10 | 52 | Male | White | 15 | High | 0 |
| 11 | 35 | Female | White | 15 | High | 0 |
| 12 | 57 | Female | White | 15+ | High | 2 |
| 13 | 35 | Male | White | 4 | High | 2 |
| 14 | 25 | Male | Asian | 1 | Low | 1 |
| 15 | 26 | Female | Asian | 2 | High | 0 |
| 16 | 53 | Male | White | 15+ | Low | 0 |
